# A Multi-Center, Multi-Parametric MRI Dataset of Primary and Secondary Brain Tumors

**DOI:** 10.1038/s41597-024-03634-0

**Published:** 2024-07-17

**Authors:** Zhenyu Gong, Tao Xu, Nan Peng, Xing Cheng, Chen Niu, Benedikt Wiestler, Fan Hong, Hongwei Bran Li

**Affiliations:** 1 Department of Neurosurgery, Second Affiliated Hospital of Anhui Medical University, Anhui Medical University, Hefei, China; 2 Department of Neurosurgery, School of Medicine, Klinikum rechts der Isar, Technical University of Munich, Munich, Germany; 3 Department of Neurosurgery, Changzheng Hospital, Naval Medical University, Shanghai, China; 4 Department of Neurosurgery, The First Affiliated Hospital of USTC, Division of Life Sciences and Medicine, University of Science and Technology of China, Hefei, China; 5 Department of Spine Surgery, Orthopedics Center of Guangdong Provincial People’s Hospital (Guangdong Academy of Medical Sciences), Southern Medical University, Guangzhou, China; 6 Department of Spine Surgery, Orthopedic Research Institute, The First Affiliated Hospital of Sun Yat-sen University; Guangdong Provincial Key Laboratory of Orthopedics and Traumatology, Guangzhou, China; 7 PET/CT center, the First Affiliated Hospital of Xi’an Jiaotong University, Xi’an, China; 8 Department of Diagnostic and Interventional Neuroradiology, School of Medicine, Klinikum Rechts der Isar, Technical University of Munich, Munich, Germany; 9 Athinoula A. Martinos Center for Biomedical Imaging, Massachusetts General Hospital, Harvard Medical School, Boston, USA

**Keywords:** Diagnostic markers, Information technology, Diagnostic markers

## Abstract

Brain metastases (BMs) and high-grade gliomas (HGGs) are the most common and aggressive types of malignant brain tumors in adults, with often poor prognosis and short survival. As their clinical symptoms and image appearances on conventional magnetic resonance imaging (MRI) can be astonishingly similar, their accurate differentiation based solely on clinical and radiological information can be very challenging, particularly for “cancer of unknown primary”, where no systemic malignancy is known or found. Non-invasive multiparametric MRI and radiomics offer the potential to identify these distinct biological properties, aiding in the characterization and differentiation of HGGs and BMs. However, there is a scarcity of publicly available multi-origin brain tumor imaging data for tumor characterization. In this paper, we introduce a multi-center, multi-origin brain tumor MRI (MOTUM) imaging dataset obtained from 67 patients: 29 with high-grade gliomas, 20 with lung metastases, 10 with breast metastases, 2 with gastric metastasis, 4 with ovarian metastasis, and 2 with melanoma metastasis. This dataset includes anonymized DICOM files alongside processed FLAIR, T1-weighted, contrast-enhanced T1-weighted, T2-weighted sequences images, segmentation masks of two tumor regions, and clinical data. Our data-sharing initiative is to support the benchmarking of automated tumor segmentation, multi-modal machine learning, and disease differentiation of multi-origin brain tumors in a multi-center setting.

## Background & Summary

High-grade gliomas (HGGs) and intracranial brain metastases (BMs) are the most prevalent malignant brain tumors in adults. They have incidence rates of 4.26 and 7–14 per 100,000 population per year, respectively^1,2^. HGG is characterized by its high malignancy due to rapid progression and spread. Recent research findings estimate that these tumors account for over 50% of malignant primary brain and central nervous system (CNS) cancers^1^. The prognosis for HGG is typically bleak, especially for the most aggressive subtype glioblastoma (GBM). Fewer than 5% of diagnosed individuals survive beyond five years. The median survival rate for newly diagnosed GBM patients is between 15 and 18 months^3^. On the other hand, BMs, which are more prevalent than primary brain tumors, also present a poor prognosis and unique diagnostic and therapeutic challenges. Median survival often only spans a few months, even with therapy^4^. The rising incidence rates can be attributed to advancements in imaging tools for detection and increased survival rates from primary malignancies^5^.

Differentiating between BMs and HGGs is clinically significant due to the contrasting surgical and therapeutic strategies^6–9^. For HGGs, a comprehensive systemic evaluation is not recommended due to the rare incidence of extracranial spread. However, for suspected brain metastatic lesions without a known systemic cancer history, it is imperative to first identify the primary malignancy and perform comprehensive systemic staging before initiating surgical or pharmacological interventions^7^. However, there are situations when no primary tumor can be found in a patient with metastases (dubbed “cancer of unknown primary”), which presents a unique challenge. While histopathological examination is currently the gold standard for definitive diagnosis^10,11^, biopsy procedures to obtain tumor tissue for this analysis can differentiate between tumor types. Nonetheless, non-invasive and rapid methods would be preferable. This is especially pertinent for patients who are not suitable for biopsy, such as those with tumors located in or near eloquent areas, or patients too debilitated to undergo biopsy or surgery^12^. In addition, it is essential to consider that the accuracy of diagnoses based on histopathology can be influenced by several factors, including the quality of the tissue sample obtained, the presence of biological heterogeneity and variety (particularly in HGGs), and the specific procedures used for processing the specimens^13–15^. Effective diagnosis hinges on the accurate integration of clinical, radiological, and histological data. Deviations or inaccuracies in the two previously mentioned factors might ultimately result in interpretative errors during the analysis of surgical specimens.

Magnetic Resonance Imaging (MRI) is the favored technique for assessing individuals with brain malignancies. Differentiating between HGGs and BMs requires considering morphological features, variety, location, and the patient’s clinical history. Challenges arise, particularly with solitary-enhancing brain lesions^7,16,17^. Intriguingly, a significant portion of BM cases present as solitary metastases, while HGGs occasionally manifest multifocal lesions^18,19^. Distinguishing between HGGs and single BMs is a significant challenge when there is a lack of documented clinical history, as these two conditions exhibit comparable radiological characteristics on MRI. Both HGGs and BMs have comparable features, including the presence of necrotic cores, uneven enhancing borders, and peritumoral edema. As a result, they commonly display identical morphological appearances on MRI scans^7^. According to existing research, it has been found that HGGs are characterized by their ability to infiltrate neighboring regions, but BMs do not possess this invasive property^20^. Subtle distinctions may arise between the two tumor types with respect to peritumoral enhancement zones, especially pertaining to the nature of edema and angiogenesis^21^. Nevertheless, conventional MRI methods continue to have challenges in accurately capturing these subtle distinctions and distinguishing between HGGs and BMs. The conventional MRI imaging sequences - FLAIR, T1-w, T1-ce, and T2-w - provide valuable information regarding tumor size, morphology, and adjacent brain structures, but they often fall short in predicting treatment outcomes or differentiating tumor subtypes^22^. As the standard of care has evolved to include more personalized treatment regimens, there is an increasing need for precise tumor differentiation and characterization.

Artificial intelligence (AI) presents a promising avenue for enhancing diagnostic accuracy^23^. Current endeavors focus on algorithms for automated lesion detection, segmentation, and differential diagnosis between HGGs and BMs^24,25^. AI can reduce human errors due to heavy workloads, thereby increasing the consistency of results. Radiomics represents an advanced technique that harnesses a multitude of features from radiographic images^26^. By quantifying a wide range of image attributes, including both conventional morphological features and intricate texture analyses, radiomics can uncover subtle imaging details that may elude human detection^26^. Studies highlight the efficacy of radiomics in assessing fundamental tumor pathophysiology and differentiating between various tumor types^26,27^.

Currently, the most comprehensive and widely used image repository for cancer imaging research is the Cancer Imaging Archive (TCIA). This archive houses imaging data for approximately 140 different types of human cancers^28^. Several databases are dedicated to gliomas, yet the TCIA only has one database specifically for BMs, comprising 156 whole-brain MRI studies^29^. Notably, there has been a recent addition of a BM database containing MRI data for 75 BM patients^30^, but it lacks data on HGG patients. This absence presents obstacles in the development of methods to differentiate between HGGs and BMs using imaging and clinical data.

Our work primarily offers multi-parametric, multi-center MRI scans and associated clinical data for patients diagnosed with both HGGs and BMs from various origins. This enriched dataset aims to facilitate the development of novel techniques for determining the origins of brain tumors. It encompasses pre-processed MRI data from 67 patients, each with unique MRI studies featuring FLAIR, T1, contrast-enhanced T1, and T2 sequences. Additionally, it includes semi-automated segmentation for all 67 lesions, leading to 67 segmentations based on both FLAIR and post-contrast T1-w sequences. Furthermore, the dataset is augmented with an extensive clinical database detailing patient demographics and treatment histories, positioning it as a valuable resource for automated tumor segmentation, disease differentiation, and the assessment of disease status for multi-origin brain tumors. Specifically, the dataset can significantly contribute to developing advanced machine-learning algorithms aimed at automated tumor segmentation. The diversity of tumor origins, despite the small sample sizes, provides a unique challenge set for developing robust algorithms that can generalize across different tumor types and origins. Furthermore, our dataset is poised to support radiogenomics research, which aims to correlate radiographic imaging features with genomic data. Although the sample sizes for some tumor origins are limited, these cases can still yield preliminary insights and hypotheses that can be further explored in larger follow-up studies. Another potential application lies in the development of personalized treatment strategies. Including clinical data alongside imaging data opens avenues for exploratory analyses that could identify imaging biomarkers predictive of treatment response or prognosis, even within the subsets of less common tumor origins. Lastly, our dataset can facilitate comparative studies between the various types of brain metastases and high-grade gliomas, contributing to a deeper understanding of their radiological distinctions and similarities. This, in turn, could aid in the differential diagnosis and treatment planning for patients presenting with these conditions.

## Methods

### Ethical approval

The dataset was retrospectively collected in accordance with the relevant ethical regulations established by the corresponding hospital’s Institutional Review Board, the Second Affiliated Hospital of Anhui Medical University (No.2023105), The First Affiliated Hospital of USTC (No. 2022-KY-242), and Changzheng Hospital (CZEC2021-068). For patients still alive, informed consent was obtained during their follow-up at the hospital, including a specific case where consent was provided by the parents of a minor. For deceased patients, consent was waived by the hospital’s ethics committee. All data utilized in this study was managed securely and de-identified to ensure the rights and privacy of the participants.

### Data description

**Fig. 1:**
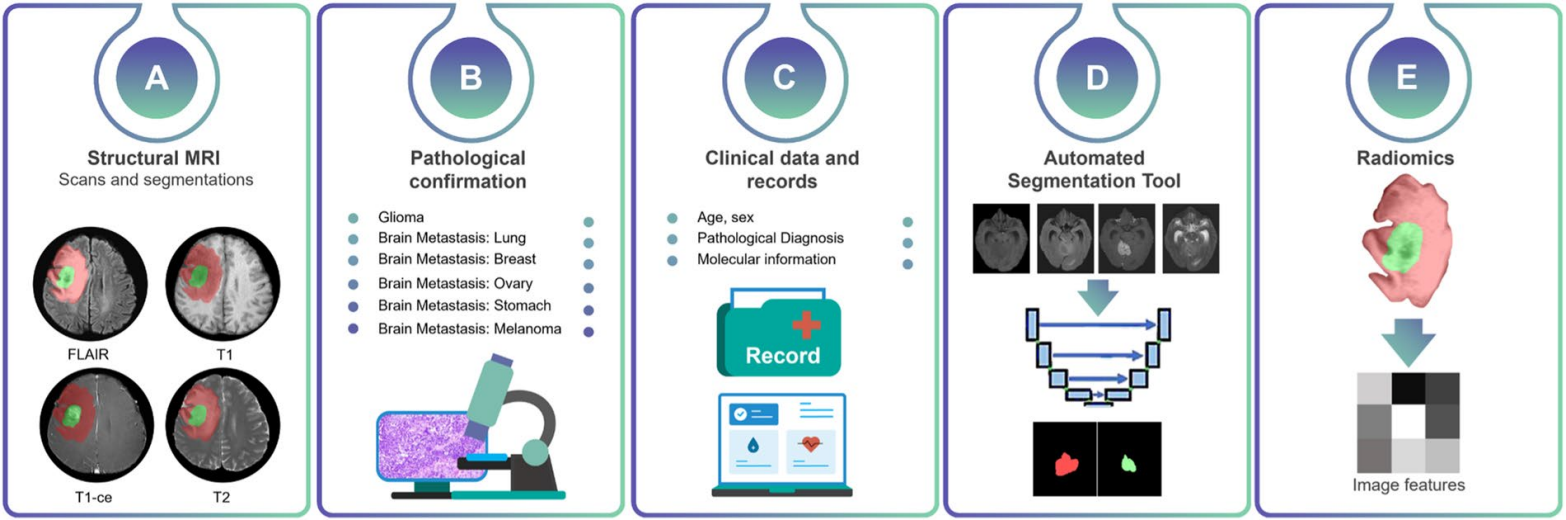
Overview of MOTUM dataset. (**A**) Structural MRIs and their derivatives including FLAIR, T1, contrast-enhanced T1, T2 and the segmentation of two tumor sub-structures. (**B**) Pathological and clinical confirmation of the tumor origin. (**C**) Clinical data and records. (**D**) Automated segmentation tool. (**E**) Radiomics feature extraction.

The multi-parametric MRI dataset for multi-origin brain tumors (MOTUM) contains 67 patients with brain tumors and provides five different sources of data, as shown in Figure 1:

1. Structural MRI scans (including DICOM files and processed images) and tumor segmentations of contrast-enhancing tumor and non-enhancing FLAIR signal abnormalities.
2. Pathological confirmation and labels specifying the origin of brain metastasis.
3. Clinical data and records.
4. Automated tumor segmentation tool.
5. Radiomics features.

### Subject characteristics

The collected data include imaging studies and clinical data of 67 HGGs and BM patients from the Second Affiliated Hospital of Anhui Medical University, Changzheng Hospital, and The First Affiliated Hospital of USTC. Inclusion criteria were defined as deceased adult patients with a pathologically confirmed diagnosis of HGGs or BMs between January 1, 2019, and January 1, 2022, availability of complete imaging studies, no noise or artifacts in the images, and availability of basic clinical data (age at diagnosis, sex, surgical result, molecular results, etc.). In addition to HGGs, which include GBM, WHO grade III and IV astrocytomas, and oligodendrogliomas (n = 29), the origins of brain metastasis were lung cancer (n = 20), breast cancer (n = 10), ovarian cancer (n = 4), gastric cancer (n = 2), and melanoma (n = 2).

### Image acquisition

MRI scans were collected as part of the routine clinical care for each patient. Scans were acquired from two vendors - Siemens or Philips 3.0 T system. The slice thickness is 5 mm.

### Pre-processing and quality control

Raw DICOM files were sorted by sequence and converted to NIfTI format. Four image sequences - FLAIR, T1, contrast-enhanced T1, and T2 - have been skull-stripped using HD-BET^31^ and rigidly co-registered with FSL^32^, using T1 as the reference. The results of skull-stripping and co-registration are visually checked. All sequences are acquired originally in a 2D manner. We opted to retain their 2D resolution instead of homogenizing them to isotropic resolutions, even though there are public tools available for this purpose^33^. Low-quality images caused by severe motion are excluded in this study. Afterward, each image is rated with two scores: mild motion (1) and no motion (2).

### Semi-automated image segmentation

Contrast-enhanced tumors (CEs) and non-enhancing abnormalities (NCEs) presented in the T1-ce and FLAIR respectively, are segmented. Initially, 30 subjects were stratified, considering their origins. The tumor signals are manually segmented with ITK-SNAP (v3.6.0)^34^. Subsequently, they are corrected by two physicians (Z.G. and T.X.) after a visual inspection slice-by-slice, using a brush tool. The rest 37 subjects are automatically segmented after training a 2D nn-UNet model^35^ on initial manual segmentations. The automated segmentations are checked and corrected by the same physicians (Z.G. and T.X.).

### Clinical data and anonymization

A comprehensive collection of clinical data was accomplished for the cohort. This dataset encompassed critical parameters such as age at diagnosis, gender, primary tumor classification, and histological subtypes, along with results from immunohistochemical staining assays. Furthermore, the dataset detailed the surgical interventions undertaken, including the scope of resection achieved. Initial anonymization of this dataset was performed at the source institutions, entailing the redaction of personally identifiable information. This was followed by the meticulous removal of private DICOM tags and any elements bearing sensitive personal data. The final step in data sanitization involved facial de-identification during the skull-stripping step, effectively precluding facial reconstruction. This rigorously anonymized dataset was then subject to a dual-review process, conducted independently by two physicians (Z.G. and F.H.), to ensure integrity and compliance with privacy standards.

### Automated image segmentation tool

After the segmentation masks for 67 subjects were obtained, automated 2D segmentation models were trained with the nnUNet framework^35^, contained to be usable in popular operating systems (MacOS, Windows, and Linux), and released. To simply the following radiomics feature extraction process based on binary segmentation masks, we train two different models for NCE and CE taking FLAIR, T1, T2, and T1-ce as the inputs. We used a 2D model instead of a 3D model considering the large slice thickness (5 mm) and relatively small sample size. We initially trained the model with 30 subjects using extensive data augmentation, applied it for 37 subjects and then manually correct the segmentation. We observed that training on 2D slides from 30 subjects can reduce significant annotation effort.

### Radiomic features

Using the PyRadiomics open-source Python library (version 2.2.0), we extracted a suite of 110 imaging features. This collection includes 16 shape-related descriptors, a range of intensity distribution metrics, and textural features linked to segmentation labels. The intensity-based features consist of basic first-order statistics along with those calculated from various matrices: 24 from the gray-level co-occurrence matrix (GLCM), 16 from the gray-level run-length matrix (GLRLM), 16 from the gray-level size-zone matrix (GLSZM), 5 from the neighboring gray-tone difference matrix (NGTDM), and 14 from the gray-level dependence matrix. Feature extraction from the pre-processed image sequences was conducted post-z-score normalization and intensity amplification by a factor of 100. Further modifications included an upward shift by 300 to maintain predominantly positive values for the first-order statistics and the application of a geometric tolerance threshold of 0.04.

## Data Records

The dataset^36^ is available on a G-Node repository and can be accessed at https://doi.gin.g-node.org/10.12751/g-node.tvzqc5. All resources can be found in a GitHub repository: https://github.com/hongweilibran/MOTUM. All files are organized with BIDS format^35^. Tumor segmentation and the corresponding have been stored in the Neuroimaging Informatics Technology Initiative (NIfTI) format, maintaining raw medical image coordinates. For each subject (e.g., */sub-0001*), the directory includes several NIfTI files containing native space FLAIR, T1-w, T1-ce, and T2 images. Their segmentation masks, radiomics features, and acquisition parameters are stored in a folder named ‘derivatives’. A general CSV file containing all the clinical data including gender, age, the origin of the tumor, the final pathological diagnosis, image quality rating, molecular information and surgery results, is created.

## Technical Validation

### Data collection

The collaborating expert board-certified neuroradiologists identified and collected the 67 HGGs and BMs patients included in the study. The tumors for each patient were pathologically confirmed and verified prior to inclusion in the study. Data curation and testing of the inclusion criteria were performed by three physicians (Z.G. T.X., and N.P.) with more than seven years’ experience in the management of medical images and then cross-checked.

### Pre-processing and segmentation method

All images after skull-stripping and co-registration were carefully checked to avoid including corrupted cases. All semi-automated segmentations performed in this study were carefully validated and corrected by experienced physicians.

### Evaluation of automated segmentation tool

**Fig. 2:**
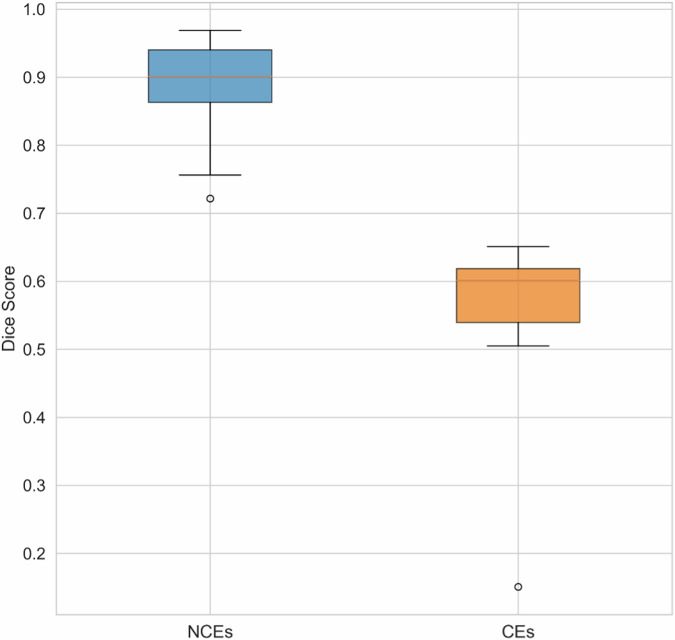
The segmentation accuracy of the automated segmentation tool in the internal evaluation set of 16 patients.

The segmentation performance of the tool was rigorously evaluated by splitting 67 patients as training and test sets based on their origin ID. Specifically, 80% patients from each category were used for training, the rest 20% were for testing. Considering its long-tail nature, at least one patient from each category was involved for testing, resulting 16 patients for the test set. Dice score which calculates the overlap between the predicted segmentation and reference segmentation, is used to quantify to segmentation. The evaluation result is shown in Fig. 2, achieving Dices scores of 0.902 and 0.587 for NCE and CE, respectively.

## Data Availability

All processing pipeline scripts are openly available. Code to generate pre-processed outputs can be accessed via https://github.com/hongweilibran/MOTUM. The automated segmentation tool can be used by following the instruction in the DockerHub page: https://hub.docker.com/repository/docker/branhongweili/motum_seg/. Pre-processing scripts for skull-stripping and co-registration are available.
